# A simple ATAC-seq protocol for population epigenetics

**DOI:** 10.12688/wellcomeopenres.15552.2

**Published:** 2021-01-07

**Authors:** Ronaldo de Carvalho Augusto, Oliver Rey, Céline Cosseau, Cristian Chaparro, Jérémie Vidal-Dupiol, Jean-François Allienne, David Duval, Silvain Pinaud, Sina Tönges, Ranja Andriantsoa, Emilien Luquet, Fabien Aubret, Mamadou Dia Sow, Patrice David, Vicki Thomson, Dominique Joly, Mariana Gomes Lima, Déborah Federico, Etienne Danchin, Aki Minoda, Christoph Grunau

**Affiliations:** 1 Univ. Montpellier, CNRS, IFREMER, UPVD, IHPE, F-66000 Perpignan and F-34095, Montpellier, France; 2 LBMC, Laboratoire de Biologie et Modélisation de la Cellule Univ Lyon, ENS de Lyon, Université Claude Bernard Lyon 1, CNRS, UMR 5239, INSERM, U1210, Lyon, 69007, France; 3 Cancer Research UK, Cambridge Institute, University of Cambridge, Cambridge, UK; 4 Division of Epigenetics, DKFZ ZMBH Alliance, German Cancer Research Center, Heidelberg, 69120, Germany; 5 Univ Lyon, Université Claude Bernard Lyon 1, CNRS, Villeurbanne, 69622, France; 6 CNRS,Station d'Ecologie Théorique et Expérimentale, Université Paul Sabatier, Moulis, 09200, France; 7 School of Molecular and Life Sciences, Curtin University, Bentley, Australia; 8 LBLGC, INRA, Université d’Orléans, Orléans, France; 9 Univ. Montpellier, CNRS, CEFE, F-34293, Montpellier, France; 10 School of Biological Sciences, University of Adelaide, Adelaide, 5005, Australia; 11 Laboratoire Evolution, Génomes Comportement, Ecologie, CNRS Université Paris Sud UMR 9191, Gif sur Yvette, 91198, France; 12 Laboratório de Malacologia, Instituto Oswaldo Cruz/Fiocruz, Rio de Janeiro, RJ, Brazil; 13 Laboratoire Évolution & Diversité Biologique (EDB UMR 5174), Université Fédérale de Toulouse; CNRS, Toulouse, 31062, France; 14 RIKEN Center for Integrative Medical Sciences, Epigenome Technology Exploration Unit, Tsurumi, Kanagawa, 230-0045, Japan

**Keywords:** epigenetics, epigenomics, ATAC-seq, Daphnia pulex, Schistosoma mansoni

## Abstract

We describe here a protocol for the generation of sequence-ready libraries for population epigenomics studies, and the analysis of alignment results. We show that the protocol can be used to monitor chromatin structure changes in populations when exposed to environmental cues. The protocol is a streamlined version of the Assay for transposase accessible chromatin with high-throughput sequencing (ATAC-seq) that provides a positive display of accessible, presumably euchromatic regions. The protocol is straightforward and can be used with small individuals such as daphnia and schistosome worms, and probably many other biological samples of comparable size (~10,000 cells), and it requires little molecular biology handling expertise.

## Introduction

Understanding the dynamic cross-talk between epigenetic mechanisms and environmental cues of animal populations is of fundamental importance for ecologists and evolutionary biologists ( Shi *et al.,* 2019). The dynamics of chromatin has long been of interest as a source of phenotypic variance within and among animal populations ( Hu & Barrett, 2017; Zhang *et al.,* 2018) and can affect their ecological performance ( Augusto *et al.,* 2019; Hawes *et al.,* 2018). In eukaryotic cells, chromatin is a dynamic structure that provides epigenetic information to control cell and gene function ( Chen & Dent, 2014). The physical organization of the chromatin landscape modulates accessibility of genomic regions and dynamically response to both external and internal stimuli. In general, accessible genomic regions are enriched in regulatory elements important for gene activity while inaccessible regions restrict binding of transcriptional regulators resulting in gene silencing ( Stergachis *et al.,* 2013). Assay for transposase accessible chromatin with high throughput sequencing (ATAC-seq) is a technique used to assess genome-wide chromatin accessibility. ATAC-seq works similarly as DNase-seq (DNase I hypersensitive sites with high-throughput sequencing) ( Song & Crawford, 2010), and determines which genomic regions are accessible to Tn5 transposase ( *i.e.* open chromatin regions), presumably the regulatory regions. Tn5 transposase inserts Illumina adapter sequences upon accessing the chromatin, which removes the need for additional steps to make the sequencing libraries later. This simple and efficient protocol reduces the starting material required, compared to DNase-seq. It also avoids many other steps such as the interaction with antibodies ( *e.g.* ChIP-seq) or chemical treatment (e.g. FAIRE-seq, WGBS) that might introduce bias. Deep sequencing of the PCR amplified Tn5 accessible regions provides a high-resolution map of accessible chromatin regions in the genome. We reasoned that this technique can not only be used to establish functional links between chromatin structure and gene function, but also to quantify epigenetic diversity in populations. This would require generation of ATAC-seq chromatin maps in single individuals. In addition, the technique should be sufficiently robust to be used by scientists who are experts in the field of population (epi)genetics and ecology, but having potentially received little training in molecular biology.

Here, we describe a streamlined and robust method for ATAC-seq of individuals of the crustacea *Daphnia pulex* and for the trematode *Schistosoma mansoni.* Our procedure is based on the protocol from Buenrostro *et al.* (2015); Corces *et al.* (2016) and Nextera DNA Library Preparation Kit (2017). Besides their ecological and epidemiological importance, both abovementioned organisms show high phenotypic plasticity in response to environmental cues ( *e.g.* the presence of predator for *Daphnia*) or during their development (schistosomes). There is a rich literature that has documented the amazing property of *Daphnia* to modify their phenotypes at the morphological, physiological, behavioral and more recently at the molecular levels in response to a large panel of environmental stressors including diet, pollution, heavy metals, and predator kairomones (reviewed in ( Harris *et al.,* 2012; Riessen, 1999)). Schistosomes also deal with a multitude of signals from the water environment as well as cues that come from their hosts, shaping morphology, metabolism, and infection success in the short-term and also their full development later in life ( Augusto *et al.,* 2017; Augusto *et al.,* 2019; Roquis *et al.,* 2018). We and others have characterized many aspects of epigenetic mechanisms behind the phenotypic plasticity of schistosomes and their cross-talk with environmental cues. Epigenetics of *D. pulex* phenotype plasticity is still poorly understood. To validate the robustness of the method, we were experimenters with different levels of expertise in molecular biology to run the experimental procedure independently using *D. pulex* and *S. mansoni*. We show here that our procedure provides robust results with individual *D. pulex* and with single adult worms of *S. mansoni*, but other organisms of similar cell number can probably also be used.

## Materials and methods

### Animal sampling

A batch of ~300 commercial *Daphnia pulex* was obtained from a commercial supplier (Aqualiment: http://www.aqualiment.eu/). For schistosomes, fresh adult worms were collected from female Swiss OF1 mice (weight mean 18g) supplied by Charles River, L’Arbresle, France. Mice had been infected by peritoneal injection with 150 mixed sexes cercaria. Water and food were given ad libitum, 12h light/dark cycle, 25°C. Housing, feeding and animal care followed the national ethical standards established in the writ of 1 February 2013 (NOR: AGRG1238753A) setting the conditions for approval, planning and operation of establishments, breeders and suppliers of animals used for scientific purposes and controls. The French Ministère de l’Agriculture et de la Pêche and French Ministère de l’Éducation Nationale de la Recherche et de la Technologie provided permit A66040 to our laboratory for experiments on animals and certificate for animal experimentation (authorization 007083, decree 87–848) for the experimenters. Hepatic perfusions were performed with lethal injection of 1 mg per kg body weight of sodium pertobartial solution (Dolehal, Vetoquinol, Lure, France) after 65 days post infection. Living adult male worm was individually transferred to a 1.5 mL Eppendorf tube and immediately processed for ATAC-seq library preparation.

### Environmental cues

**Figure 1.**
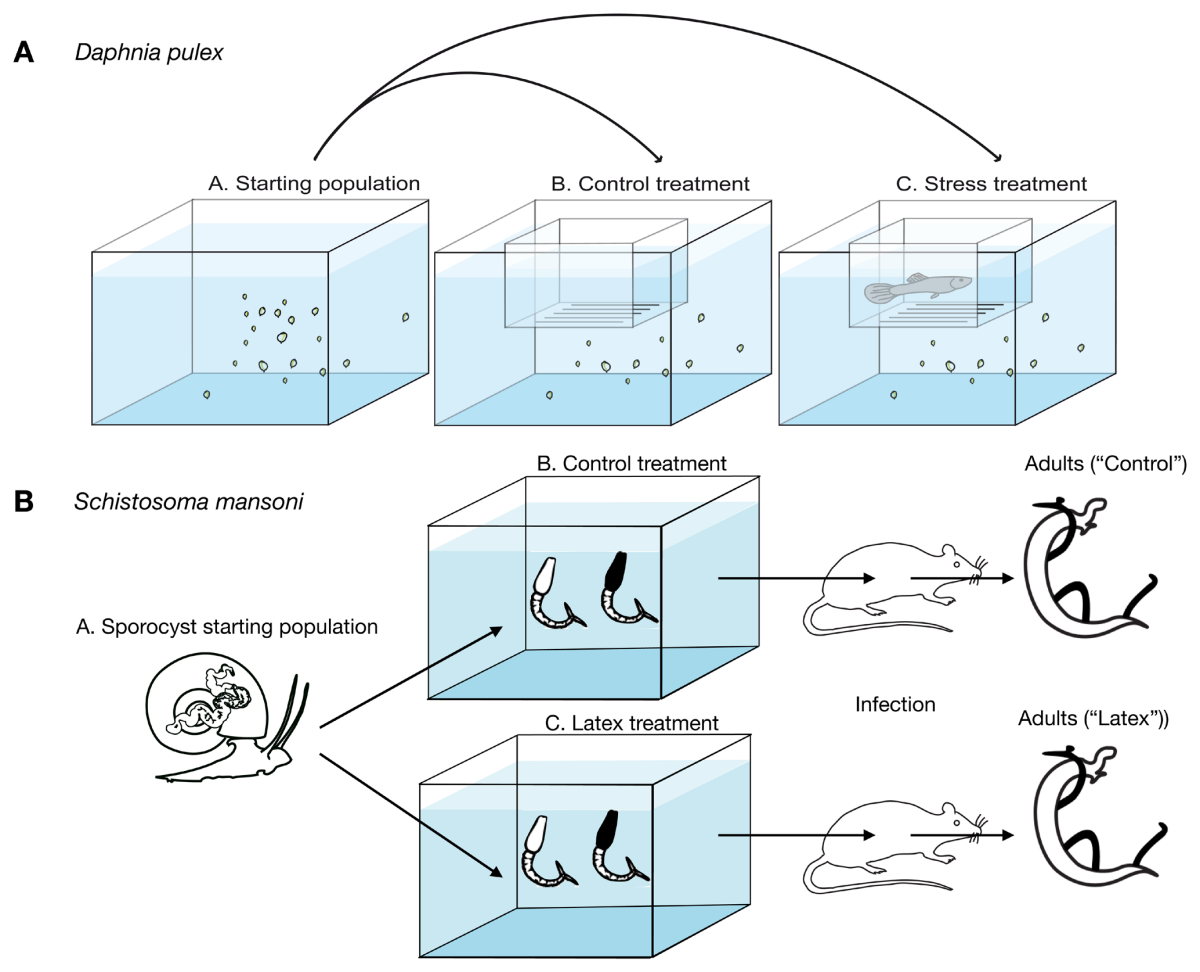
Schematic representation of experimental design. ( **A**) *Daphnia* were put into a water tank and allowed to acclimate (start population). Then, two experimental tanks were set up following strictly the same design. The only difference was the presence of a predator (a guppy trained to eat daphnia) in the floating plastic fish breeding isolation box in the stress treatment. **(B)** *S.mansoni* infected snails were used to produce cercariae that were separated into two populations, one treated with Latex in well water, the other without treatment. After one hour cercaria were used to infect mice. Adult worms were recovered at 65 days post-infection by perfusion and used for ATAC-seq.

At their arrival, *D. pulex* were immediately split into two sets of equal size (~ 150 × 2) and placed in two independent experimental tanks ( *i.e.* initial density of 75 ind/L), hereafter called the ‘stress’ and the ‘control’ tanks. Each experimental tank consisted in a 2-L plastic aquaria (L × l × h = 18 ×12 × 11 cm) supplied with clean water, inside of which a floating plastic fish breeding isolation box (L × l × h = 12.5 × 8 ×7 cm) was placed. These isolation boxes are transparent with a series of 1 mm cracks on the bottom wall to allow water connection between the tanks and inside the isolation box. *D. pulex* were acclimated in their respective experimental tanks out of the isolation box for 20 days prior to starting the experiment. This lag time before the experiment also allowed the production of new *D. pulex* offspring born in our experimental setup. During this acclimating period, only negligible mortality was observed and newly hatched *D. pulex* were observed in the two experimental tanks. After this 20-day acclimating period, a predator ( *i.e.* a guppy fish previously trained to eat *D. pulex*) was introduced into the isolation box of one experimental tank during 15 days ( *i.e.* hereafter called the ‘stress treatment’, compared to the ‘control treatment’) ( Figure 1A). During the experiment the fish was fed every other day with 10 *D. pulex* collected alternatively from the stress and the control tank (i) to avoid subsequent biases in density between the experimental treatments and (ii) to account for a possible effect of *D. pulex* sampling on congeners‘ responses. *D. pulex* sampling for fish feeding was achieved using a sterile 3-ml plastic transfer pipet. This experimental setup allowed the *D. pulex* of the stress treatment to experience an indirect predation pressure ( *i.e.* without predation risk) through a direct visual contact with the predator and an olfactory contact with environmental cues released by the predator. Over the experiment, the *D. pulex* and the predator were maintained at room temperature following the natural photoperiod and the former were fed ad libitum with clean phytoplancton ( *i.e. Chlorella sp*.) reared in our lab facilities. Living *D. pulex* were sampled by pipetting through a 1 mL automatic pipette with enlarged openings of the pipetting tips. To avoid experimenter bias, 13 different persons sampled at least one individual each. Finally, each specimen was then individually transferred to a 1.5 mL Eppendorf tube and was immediately processed for ATAC-seq library preparation as follows. Controls were done without organisms as input.

For schistosomes, treatment of vertebrate infective larvae (cercariae) and recovery of adult worms was done as described in Augusto *et al.,* 2017; briefly, *E*. *milii* var. *hislopii* latex was collected at Ilha do Governador district (22°48´09´´S/43°12´35´´W), Rio de Janeiro, Brazil lyophilized at -52°C on 8 ×10 ^-1^ mBar for three 12-hour cycles in a Modulyo 4K Freeze Dryer with an acrylic chamber (Edwards High Vacuum Int., UK). The dose of the powdered lyophilized latex used to expose cercariae was 1.4 mg/L, described by Augusto *et al.,* 2017 as LC _50_ for the intermediate host *Biomphalaria glabrata*. Cercariae were collected from infected host *B. glabrata* and split into two groups as follows: one group described as ‘Control treatment’ ( Figure 1B) was kept in a tank of water for one hour while other group described as ‘Latex treatment’ was exposed to a solution of *E*. *milii* lyophilized latex in distilled water (1.4 mg/L), both for one hour. We infected 10 female mice (4 weeks-old Swiss-Webster mice, weight mean 18 g) with 150 exposed cercariae per mouse and another 10 mice were infected with 150 mock treated cercariae per mouse, all using standard percutaneous inoculation and mixed sexes. Finally, parasites couples were recovered at 65 days post-infection by perfusion. ( Figure 1B). Males were manually separated from females and only male worms were used.

### Counting of nuclei

Individual male *S. mansoni* worms and individual *D. pulex* were transferred into 1.5 ml Eppendorf tubes and all excess liquid was removed. Animals were resuspended in 20 – 100 µL of Nuclei EZ lysis buffer (Sigma N3408-200ml) and grinded with disposable polypropylene pestles. Two µL were applied on microscope glass slides and 2 µL ProLong Diamond Antifade mountant with DAPI (Invitrogen P36966) were added to stain nuclei. Nuclei in the total volume were counted under a Zeiss fluorescence microscope. 

### Transposase mixture

The necessary material is listed in Table 1 and Table 2 and must be prepared in advance. In addition, nuclease-free water, high fidelity DNA polymerase for PCR and corresponding buffers, freshly prepared 80% ethanol, refrigerated centrifuge, 0.2 ml PCR tubes, 1.5 ml tubes, ThermoMixer with agitation, PCR thermal cycler, qPCR instrument, magnetic rack, 1 mL pipette, 100 μL pipette, and 10 μL pipette are needed.

**Table 1.** Externally sourced materials.

Item name	Vendor	Catalog ID
1% Molecular biology-grade IGEPAL CA-630	Sigma-Aldrich	I8896
2xTD (Tagment DNA buffer from Nextera kit)	Illumina	FC-121-1030
TDE1 (Tagment DNA Enzyme from Nextera kit)	Illumina	FC-121-1030
QIAquick PCR Purification Kit	Qiagen	28104
AMPure XP beads	Agencourt	A63880
Bioanalyzer High-Sensitivity DNA Analysis kit	Agilent	5067-4627
10,000X SYBR I	Invitrogen	S-7563

**Table 2.** Reagents produced in the laboratory.

Phosphate buffered saline (PBS)	137mM NaCl; 27mM KCl; 100mM Na _2_HPO _4_; 18mM KH _2_PO _4_
2× Tagmentation buffer (2× TD buffer)	20 mM Tris(hydroxymethyl)aminom ethane; 10 mM MgCl _2_; 20% (vol/vol) dimethylformamide ( Wang *et al.,* 2013)

An Eppendorf ThermoMixer was then set with agitation to 37°C and the following steps performed.

- For *D. pulex* remove all water by pipetting with 100 µL tip;

or

Perfuse *S. mansoni* worms and take single worm as dry as possible with forceps

- Wash once with 50 μL of cold 1x PBS buffer and remove all supernatant by pipetting, being careful not to remove your sample;

- Add to each sample
  ○ 25 μL 2× TD buffer
  ○ 2.5 μL TDE1
  ○ 0.5 μL 1% IGEPAL
  ○ 22 µL Nuclease-free water

This gives 50 µL of transposase mixture for each sample. The samples are pipetted up and down 10 times to disrupt cells. In our hands, the addition of IGEPAL directly into the tagmentation reaction, and the agitation eliminated the need for a separate cell lysis step and streamlined the protocol. 

### Chromatin tagmentation

This step uses the Nextera Tn5 transposome to ‘tagment’ the chromatin, which is a process that fragments the chromatin and tags the DNA with adapter sequences in a single step.

- Tagmentation reactions are incubated at 37°C for 30 min in an Eppendorf ThermoMixer with agitation at 300 rpm.
- Tagmented chromatin is immediately purified using a QIAGEN MinElute Reaction Cleanup kit or a QIAquick PCR Purification Kit , and purified DNA is eluted into 20 μL of elution buffer (10 mM Tris-HCl, pH 8).

### Library amplification

This step amplifies the tagmented DNA using a limited-cycle PCR program. PCR is carried out with a universal index Ad1 and an index (barcode) primer Ad2, as described in Table 3 ( Buenrostro *et al*., 2015). Two library amplification methods were tested and validated in our hands as follows:

**Table 3.** PCR Primer ID and sequence.

Index ID	Sequence
Ad1_noMX:	AATGATACGGCGACCACCGAGATCTACACTCGTCGGCAGCGTCAGATGTG
Ad2.1_TAAGGCGA	CAAGCAGAAGACGGCATACGAGATTCGCCTTAGTCTCGTGGGCTCGGAGATGT
Ad2.2_CGTACTAG	CAAGCAGAAGACGGCATACGAGATCTAGTACGGTCTCGTGGGCTCGGAGATGT
Ad2.3_AGGCAGAA	CAAGCAGAAGACGGCATACGAGATTTCTGCCTGTCTCGTGGGCTCGGAGATGT
Ad2.4_TCCTGAGC	CAAGCAGAAGACGGCATACGAGATGCTCAGGAGTCTCGTGGGCTCGGAGATGT
Ad2.5_GGACTCCT	CAAGCAGAAGACGGCATACGAGATAGGAGTCCGTCTCGTGGGCTCGGAGATGT
Ad2.6_TAGGCATG	CAAGCAGAAGACGGCATACGAGATCATGCCTAGTCTCGTGGGCTCGGAGATGT
Ad2.7_CTCTCTAC	CAAGCAGAAGACGGCATACGAGATGTAGAGAGGTCTCGTGGGCTCGGAGATGT
Ad2.8_CAGAGAGG	CAAGCAGAAGACGGCATACGAGATCCTCTCTGGTCTCGTGGGCTCGGAGATGT
Ad2.9_GCTACGCT	CAAGCAGAAGACGGCATACGAGATAGCGTAGCGTCTCGTGGGCTCGGAGATGT
Ad2.10_CGAGGCTG	CAAGCAGAAGACGGCATACGAGATCAGCCTCGGTCTCGTGGGCTCGGAGATGT
Ad2.11_AAGAGGCA	CAAGCAGAAGACGGCATACGAGATTGCCTCTTGTCTCGTGGGCTCGGAGATGT
Ad2.12_GTAGAGGA	CAAGCAGAAGACGGCATACGAGATTCCTCTACGTCTCGTGGGCTCGGAGATGT
Ad2.13_GTCGTGAT	CAAGCAGAAGACGGCATACGAGATATCACGACGTCTCGTGGGCTCGGAGATGT
Ad2.14_ACCACTGT	CAAGCAGAAGACGGCATACGAGATACAGTGGTGTCTCGTGGGCTCGGAGATGT
Ad2.15_TGGATCTG	CAAGCAGAAGACGGCATACGAGATCAGATCCAGTCTCGTGGGCTCGGAGATGT
Ad2.16_CCGTTTGT	CAAGCAGAAGACGGCATACGAGATACAAACGGGTCTCGTGGGCTCGGAGATGT
Ad2.17_TGCTGGGT	CAAGCAGAAGACGGCATACGAGATACCCAGCAGTCTCGTGGGCTCGGAGATGT
Ad2.18_GAGGGGTT	CAAGCAGAAGACGGCATACGAGATAACCCCTCGTCTCGTGGGCTCGGAGATGT
Ad2.19_AGGTTGGG	CAAGCAGAAGACGGCATACGAGATCCCAACCTGTCTCGTGGGCTCGGAGATGT
Ad2.20_GTGTGGTG	CAAGCAGAAGACGGCATACGAGATCACCACACGTCTCGTGGGCTCGGAGATGT
Ad2.21_TGGGTTTC	CAAGCAGAAGACGGCATACGAGATGAAACCCAGTCTCGTGGGCTCGGAGATGT
Ad2.22_TGGTCACA	CAAGCAGAAGACGGCATACGAGATTGTGACCAGTCTCGTGGGCTCGGAGATGT
Ad2.23_TTGACCCT	CAAGCAGAAGACGGCATACGAGATAGGGTCAAGTCTCGTGGGCTCGGAGATGT
Ad2.24_CCACTCCT	CAAGCAGAAGACGGCATACGAGATAGGAGTGGGTCTCGTGGGCTCGGAGATGT

***Option 1** (for Promega GoTag G2).* Combine the following in a PCR tube for each sample: 9.5 μL Nuclease-free MilliQ water; 20 μL Purified transposed DNA; 10 μL 5x GoTaq G2 buffer; 4 μL MgCl _2_; 2.5 μL Universal Ad1_noMX primer (25 µM); 2.5 μL Specific Index primer Ad2.*, different for each sample (25 µM); 1 μL dNTPs (10 mM); 0.5μL GoTaq G2.

Or

***Option 2** (for NEB mix, more convenient but more expensive).* Combine the following in a PCR tube for each sample: 20 μL purified transposed DNA; 2.5 μL Universal Ad1_noMX primer (25µM); 2.5 μL Specific Index primer Ad2.*, different for each sample (25µM); 25 μL NEBNext High-Fidelity 2X PCR Master Mix

In both options the final volume is 50 µL. The samples are pre-amplified using a PCR machine with the program described in Table 4.

**Table 4.** PCR program for library pre-amplification.

Step	Temp	Duration	Cycles
Pre-Warming	72°C	5 min	1
Initial denaturation	98°C	30 sec	1
Denaturation	98°C	10 sec	5
Annealing	63°C	30 sec	
Extension	72°C	1 min	
HOLD	12°C	∞	1

In order to reduce GC and size bias in the subsequent PCR, the PCR dynamics is monitored using qPCR to stop amplification prior to saturation. To run a qPCR side reaction, we combined the following depending on the option that had been chosen previously:

**Option 1**: 5 μl PCR product of the initial pre-amplification reaction (keep the remaining 45 µL at 4°C); 2.5 μl 5x GoTaq G2 buffer; 0.1 μL GoTaq 2; 3.14 μl Nuclease-free MilliQ water; 0.25 μL Universal Ad1_noMX primer (25µM); 0.25 μL Ad2.* indexing primer (25µM) ;1 μL MgCl _2_; 0.25 μL dNTPs; 0.1 μL 100X SYBR I

or

**Option 2**: 5 μl PCR product of the initial pre-amplification reaction (keep the remaining 45 µL at 4°C); 4.41 μL Nuclease-free MilliQ water; 0.25 μL Ad1_noMX primer (25 μM); 0.25 μL Ad2.* indexing primer (25 μM); 0.09 μL 100X SYBR I; 5 μL NEBNext High-Fidelity 2X PCR MasterMix

The samples are amplified in a qPCR machine with the program set out in Table 5.

**Table 5.** PCR program for library amplification.

Step	Temp	Duration	Cycles
Initial denaturation	98°C	30 sec	1
Denaturation	98°C	10 sec	20
Annealing	63°C	30 sec	
Extension	72°C	1 min	
HOLD	12°C	∞	1

**Figure 2.**
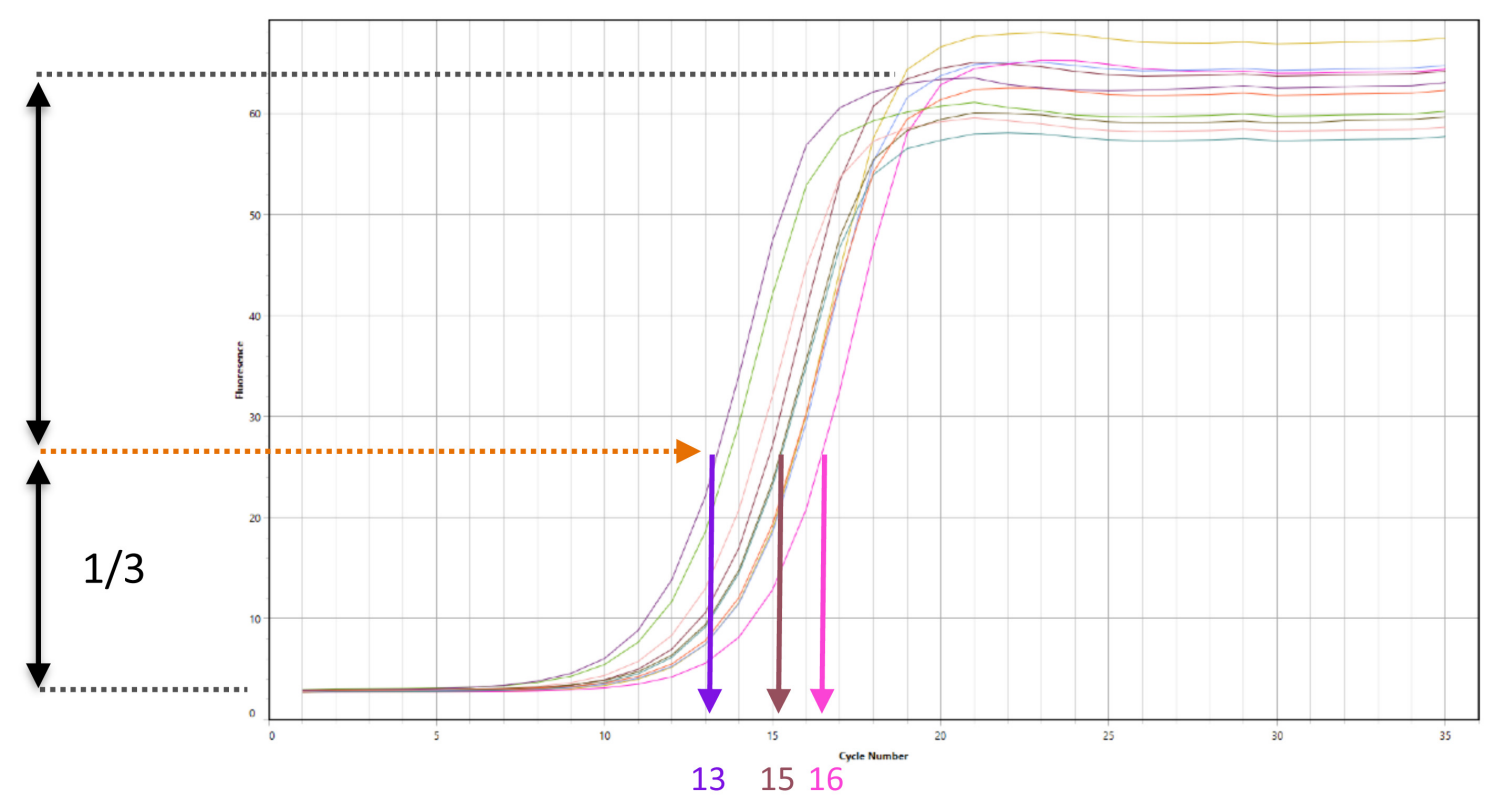
Example qPCR amplification profile. (X-axis) Number of PCR cycles. (Y-axis) Fluorescence intensity. One-third of the maximum fluorescent intensity is shown by the orange line and the optimal number of additional cycles to perform are indicated for three example ATAC-seq libraries.

To calculate the optimal additional number of cycles needed for the remaining 45 μL PCR, relative fluorescence is plotted against cycle number and the cycle number that corresponds to one-third of the maximum fluorescent intensity is determined ( Figure 2). In our experience, the total number of amplification cycles must not exceed 21 ( Augusto *et al.,* 2019).

The remaining 45 μL PCR reaction is run with the additional number of cycles and purified with a QIAGEN MinElute Reaction Cleanup kit or a QIAquick PCR Purification Kit, or similar, and eluted into a total of 45 μL of elution buffer (10 mM Tris-HCl, pH 8). Elution can be done in two rounds.

**Figure 3.**
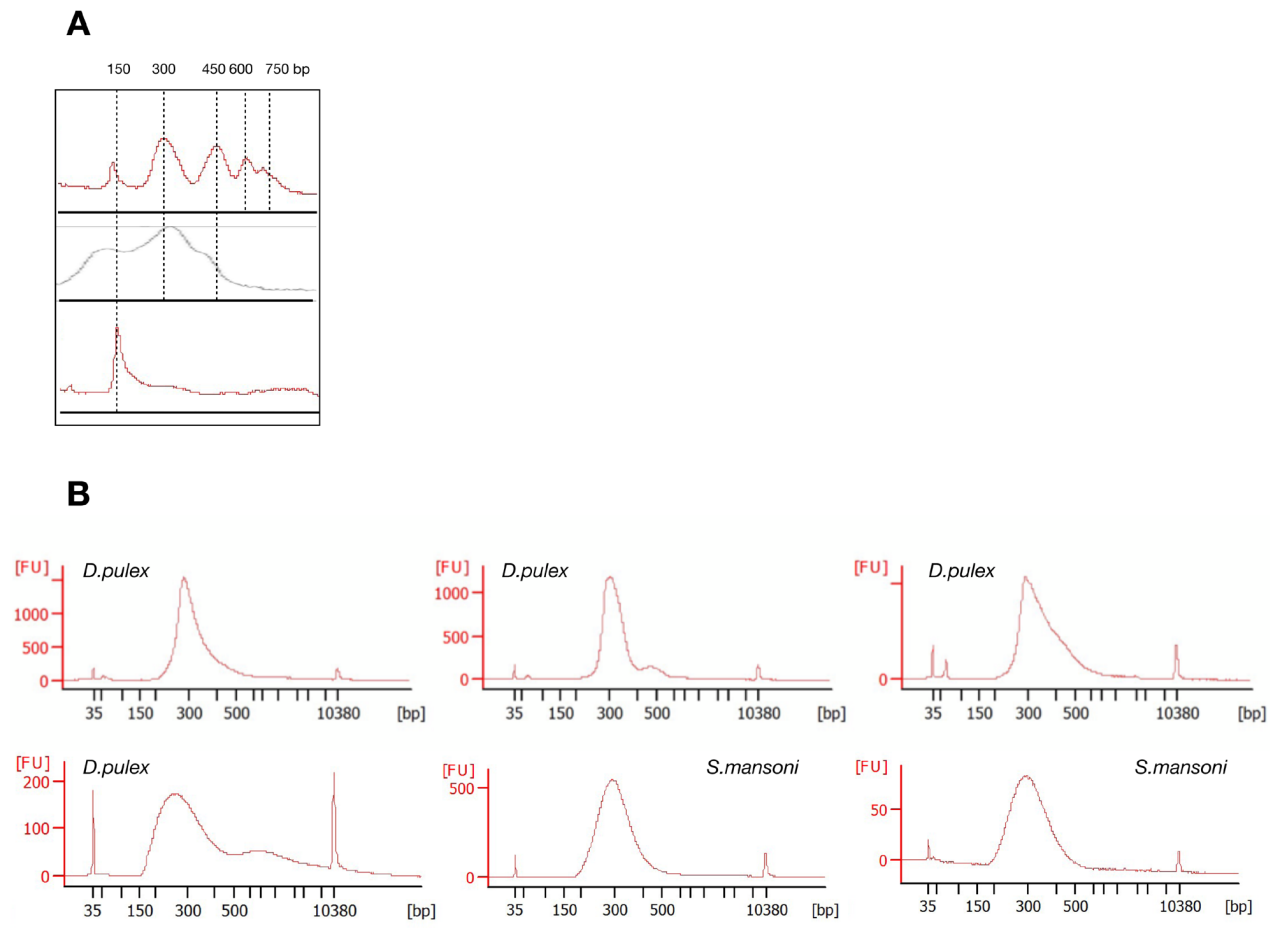
Examples of fragmentation profiles of ATAC-seq libraries before ( **A**) and after size selection ( **B**). X-axis: Base pairs. Y-axis: Fluorescence intensity. ( **A**) Peaks correspond to nucleosome-free region, mono- to tetra-nucleosome fractions. Bottom lane: too strong fragmentation, thus Tn5 quantity needs to be decreased. ( **B**) Examples of BioAnalyser profiles of ATAC-seq libraries after size selection. Fragment size should be between 150 and 800 bp. Peaks at 35 bp and 10380 bp are spiked-in marker peaks for the BioAnalyser.

Fragments are separated by electrophoresis through a 1.5% agarose gel or on a Bioanalyzer chip. A ladder that corresponds to the nucleosome-free region and multiple nucleosome-size fragments should be seen (one nucleosome = about 150 bp). A single band at around 150 bp indicates sample degradation or over-fragmentation. Ideally, five bands should be obtained, three bands are acceptable ( Figure 3A).

### AMPure XP beads double-side purification

This step enriches for the nucleosome-free (~300 bp) as well as di and tri-nucleosome fragments. Removing small fragments (primer dimers) is important for optimal sequencing. First transfer 45 µL to an Eppendorf tube (or use PCR tube directly), add 22.5 μL (0.5X original volume, to remove large fragments) AMPure XP beads, pipet up and down 10 times to mix thoroughly. Incubate at room temperature for 10 minutes and place tubes in magnetic rack for 5 minutes. Transfer supernatant to new tube and add 58.5 μL (1.3X original volume, to remove small fragments) AMPure XP beads, pipet up and down 10 times to mix thoroughly. Incubate at room temperature for 10 minutes, place tubes in magnetic rack for 5 minutes and discard supernatant. Wash beads with 200 μL 80% ethanol (freshly made), pipet ethanol over beads 10 times, then discard ethanol. Ensure all ethanol is removed. Leave tube on magnetic rack with cap open for 3 to maximum 10 minutes depending on ambient humidity. The beads should be ‘glowing’ but not wet. Be careful not to over-dry them, which will decrease elution efficiency. Resuspend beads in 20 μL nuclease-free water, pipet up and down 10 times to mix thoroughly, place tube in magnetic rack for 1–5 minutes and transfer supernatant to new tube. This step can be replaced by Diagenode IP-Star, size selection 320 bp.

We have not systematically investigated if different purification procedures influence on the result. Purified libraries should be stored at -20°C and can be used for sequencing after up to 4 months.

### Libraries check

Size profiling can be performed using an Agilent Bioanalyzer High Sensitivity DNA Assay. Expected profiles are shown in Figure 3B. Bioanalyzer profiles or KAPA library quantification kit were used to quantify libraries and proceed to sequencing. We present here data sequenced on a NextSeq550 High Output Flowcell as paired-end and 75 bp.

### Detection of chromatin structure differences

Sequence quality was checked with FastQC ( http://www.bioinformatics.babraham.ac.uk/projects/fastqc/). For *D. pulex*, reference genome was downloaded from ftp://ftp.ensemblgenomes.org/pub/metazoa/release-40/fasta/daphnia_pulex/dna/Daphnia_pulex.V1.0.dna.toplevel.fa.gz, corresponding to GenBank assembly accession GCA_000187875.1. For *S. mansoni*, v5 reference genome was downloaded from ftp://ftp.sanger.ac.uk/pub/pathogens/Schistosoma/mansoni/Archive/S.mansoni/genome/Assembly-v5/. For both, alignment was done with Bowtie2 evoking the following parameters: bowtie2-align-s basic-0 -p 6 -x genome -N 1 -L 32 -i S,1,1.15 --n-ceil L,0,0.15 --dpad 15 --gbar 4 --end-to-end --score-min L,-0.6,-0.6. Uniquely aligned reads were retained by filtering the tag “XS:i:” that is absent in their alignement annotations.

For visualisation of ATAC profiles, all BAM files from *D. pulex* or *S. mansoni* were merged, converted to header-free SAM, and downsampled with a custom script that draws random lines to the condition with the lowest number of aligned reads (409,000 aligned reads for *D. pulex* and 15,000,000 reads for *S. mansoni*). For both, PCR duplicates were removed with SamTools RmDup. Bedgraph files were generated with MACS2 and/or ChromstaR, lower fold bound of 5, upper fold bound 50, band width 300 bp, minimum FDR for peak detection of 0.05 and without calling broad regions. Bedgraphs were loaded into IGV for visual inspection. For analysis of individual *D. pulex* or *S. mansoni*, background correction was done with MACS bdgcmp and linear scale fold enrichment (--method FE). Bedgraph was converted into BigWig. The DeepTools suite was used for representation of metagene profiles based on the forward strand for both organisms. For *Daphnia*, gene annotation files were downloaded from ftp://ftp.ensemblgenomes.org/pub/metazoa/release-40/fasta/daphnia_pulex/cds/Daphnia_pulex.V1.0.cds.all.fa.gz. More information is available at https://metazoa.ensembl.org/Daphnia_pulex/Info/Annotation/. For *S. mansoni*, gene annotation was downloaded from ftp://ftp.sanger.ac.uk/pub/pathogens/Schistosoma/mansoni/Archive/S.mansoni/genome/Assembly-v5/ and modified to contain only genes and pseudogenes (sma_v5.2_2015.01.06_genes_pseudogenes_rnas.gff3). Two different approaches were used for further data analysis. One uses a combination of peakcalling with MACS2, extraction of read coverage in peaks with BEDtools intersect intervals with A-File the MACS peaks and B-File the uniquely aligned BAM, and DESeq2 for differential analysis. To detect all peak regions for all conditions, For *Daphnia*, BAM files of control and stress conditions were merged and peakcalling was performed with MACS2 as described above. The number of reads overlapping peak regions was extracted with bedtools intersect -a peakfile.bed -b individual_bam_files.bam -header -wa -c, Columns 4 and 11, corresponding to peak-names and number of overlapping features, *i.e.* coverage were used as input for DESeq2. All analyses were done at the galaxy instance of the Labex CeMEB/IHPE ( http://bioinfo.univ-perp.fr).

The second approach was based on Hidden-Markow-Models (HMM) implemented in ChromstaR (v.1.2.0) for genome-wide characterization of open chromatin landscape. On this approach control and stress condition were processed in two steps: (1) we fitted a univariate HMM over each ATAC-seq samples individually and (2) we performed a multivariate HMM over the combined ATAC-seq samples in each condition. For that, BAM files were processed under the differential mode, with a false discovery rate (FDR) cutoff of 0.05 and bin size of 500.

## Results

### The method can be used by scientists with low expert level in molecular biology

The protocol described in the methods section was tested by 13 experimenters with molecular biology expert level ranging from untrained to over several 10 years of experience, or some who had retired from active wet-bench work several years ago. In only two cases ATAC-seq library production did not succeed. ********

### ATAC-Seq can be used on individual Daphnia and individual Schistosoma adults

**Figure 4.**
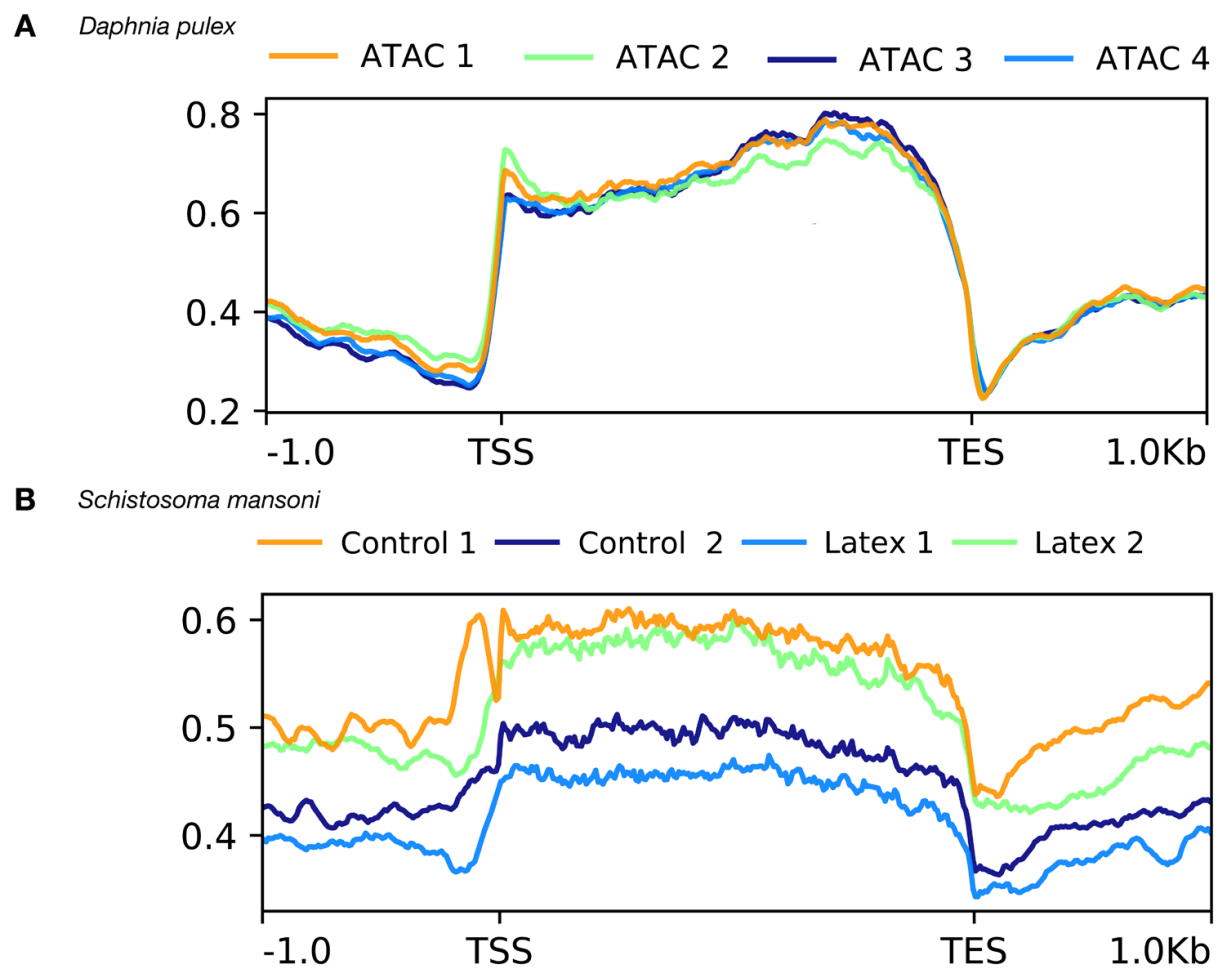
Superposed metagene ATAC profiles of four individuals after sequencing and analysis. X-axis in base-pairs. TSS = Transcription start site, TES = transcription end site. Y-axis average enrichment of ATAC-seq reads over genes and upstream and downstream regions. Enrichment of accessible chromatin occurs along the entire length of the genes. The metagene profiles are almost identical for *Daphnia* ( **A**), while there is more heterogeneity of the profiles in *schistosomes* ( **B**). Nevertheless, the profiles are in both cases very reproducible indicating the robustness of the ATAC-seq procedure.

Our ATAC-seq procedure delivered reproducible chromatin profiles for individual *D. pulex* and adult *S. mansoni*. Projection of ATAC-seq reads on a metagene profile indicated that Tn5 accessible and thus presumably open chromatin structure occurs at the TSS and in gene bodies ( Figure 4). Individual daphnia contain 8,500–10,000 nuclei and adult male schistosomes 18,000 – 20,000 nuclei.

### Exposure to predator cues leads to morphological differences in Daphnia

**Figure 5.**
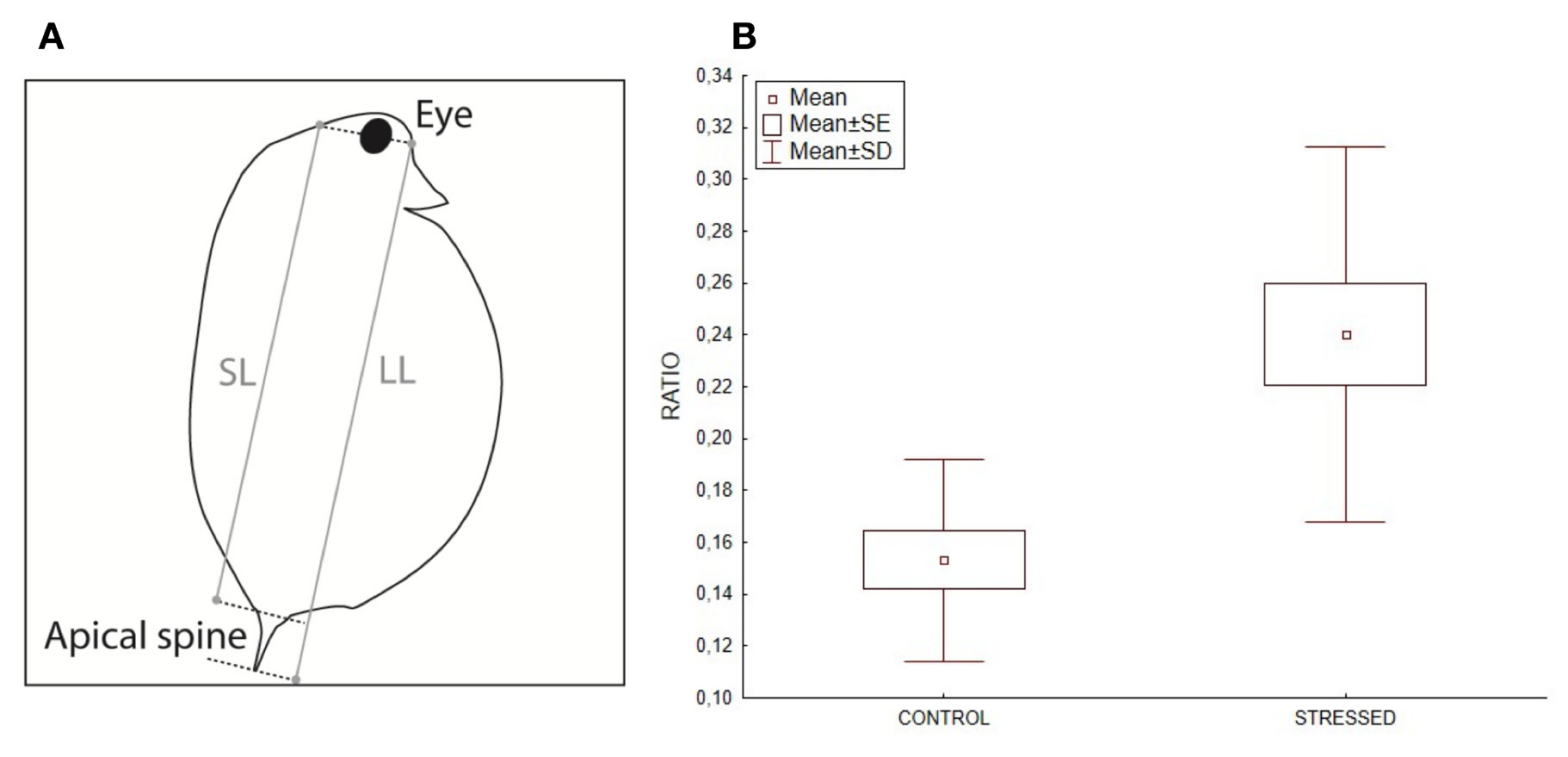
( **A**) Schematic representation of the measures taken on daphnia. SL = short length, LL = Long length. ( **B**) Boxplot of morphometric ratios of (LL-SL)/SL in control and stressed *daphnia* populations (control: n = 12; stress: n = 14).

Our results show that on average, the (LL-SL)/SL ratio calculated for *D. pulex* from the stress treatment (N = 14; Mean = 0.24 ± 0.072) was significantly higher than that from the control treatment (N = 12; Mean = 0.15 ± 0.039; Mann-Whitney U Test, *U* = 19, Z = -3.32, P < 0.001) ( Figure 5). This result confirms the expected induction of anti-predatory morphs in the stress treatment. It is noteworthy that the quantified morphological response to predation pressure observed in the stress treatment most likely reflects a more general response of stressed *D. pulex* including morphological, physiological and behavioural changes ( Boersma *et al.,* 1999) Our first intention in comparing *D. pulex* from the two experimental treatments was to confirm that we effectively induced a global response in stressed individuals, these responses having been otherwise much better documented previously ( Riessen, 1999).

### Exposure to predator cues leads to differences in chromatin structure between exposed (stressed) and unexposed (control) Daphnia pulex

**Figure 6.**
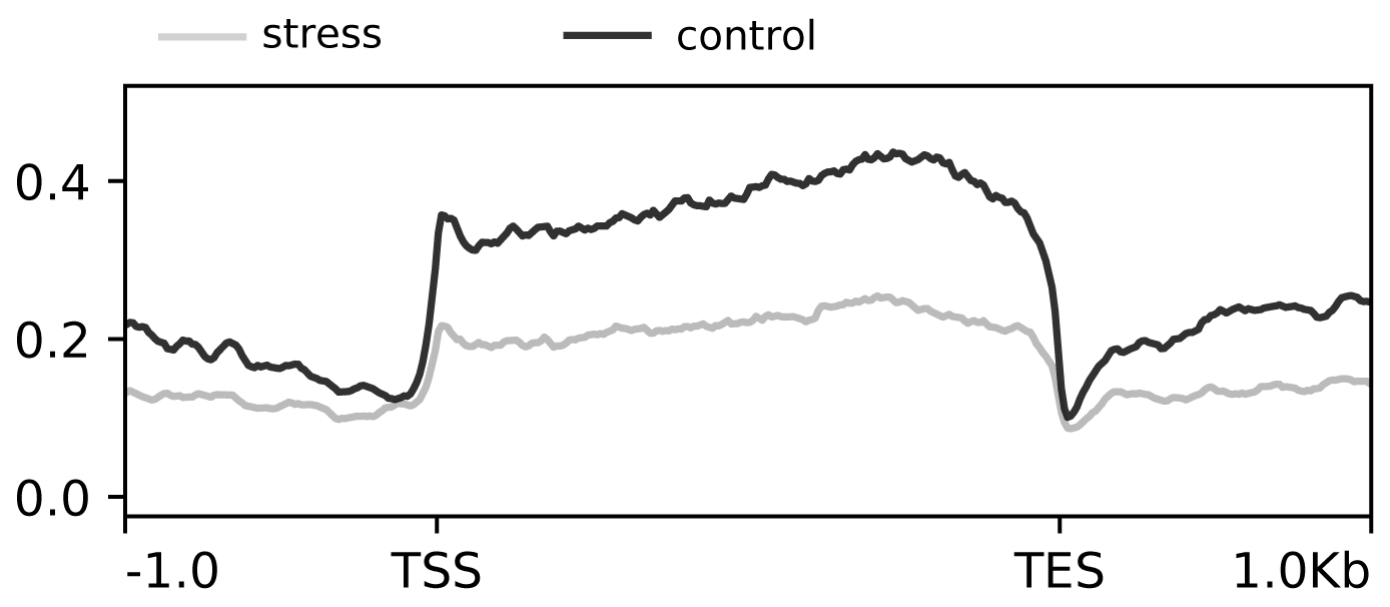
Combined metagene ATAC profiles of stressed and control *daphnia* populations. X-axis in base-pairs. TSS = Transcription start site, TES = transcription end site. Y-axis average enrichment of ATAC-seq reads over genes and upstream and downstream regions.

Using the DESeq2 procedure described above for ‘start’ vs. ‘control’ we identified 66,194 differences between ‘control’ and ‘stressed’. This is by far too many, and indeed, shifts in MA plots (not shown) indicated that the assumption that is underlying the algorythm used in DESeq2 and the requires that most sites do not change, was violated. Metagene profiles, using the same number of aligned reads over the entire genome, lend further support to the finding that ‘stressed’ samples had on average fewer reads over genes than ‘control’ samples indicating major changes in chromatin structure ( Figure 6).

**Figure 7.**
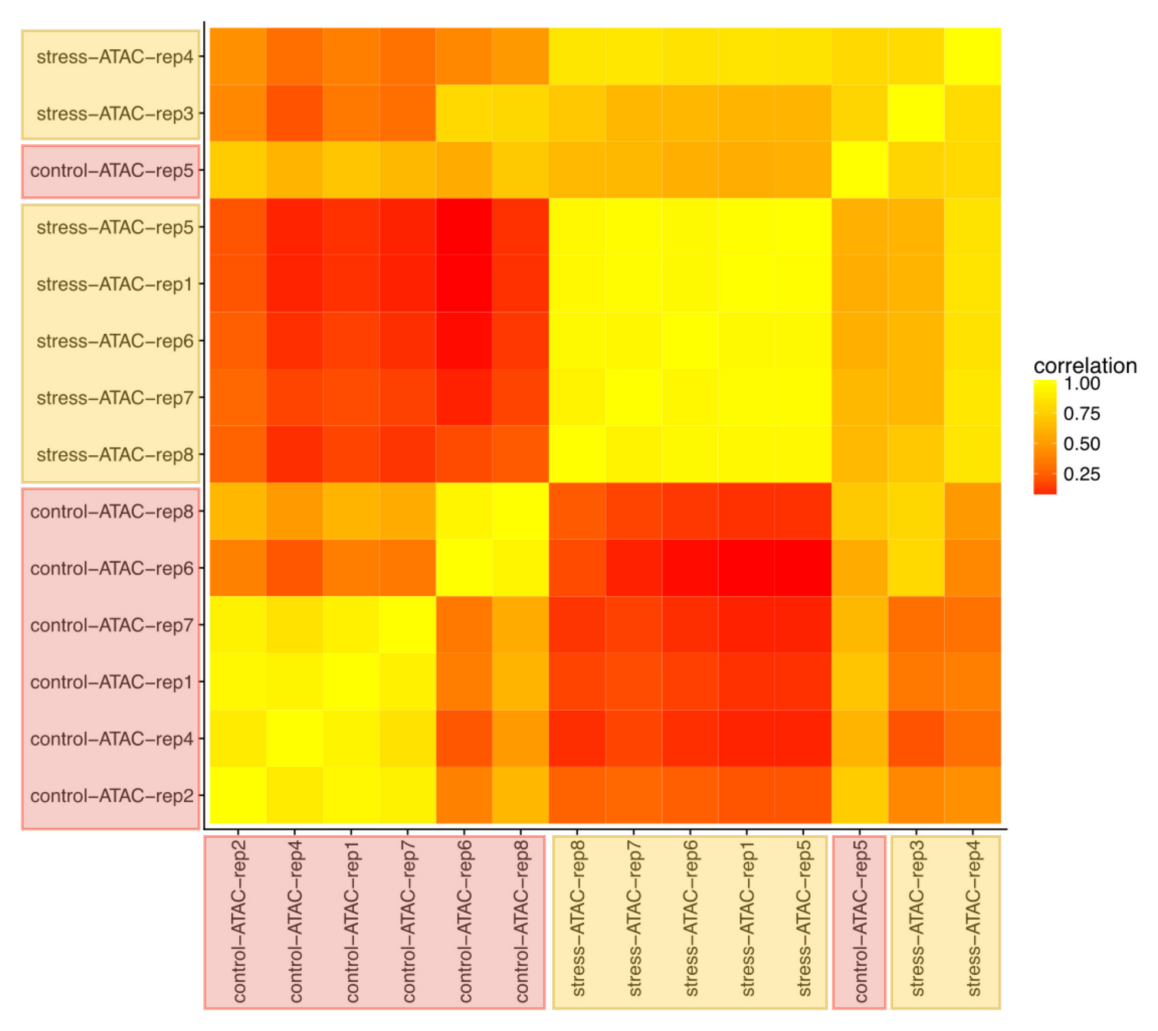
Clustering of individual *daphnia* based on their ATAC-seq profiles. Heat map indicating similarity in the HMM ChromstaR results. Generally, samples from the stressed and the control populations each cluster together.

This also means that there is a large number of regions for which no reads could be recovered in the stressed samples. This is not due to a general lower accessibility of Tn5 to the cells and nuclei because of a thicker cuticle or a similar phenotypic trait because the insert size distribution of start, control and stressed populations are similar (Supplementary file 1). If DNA was more inaccessible in the stressed population we would expect longer fragments. To cope with the general decrease of ATAC-Seq reads in the stressed population, we resorted to ChromstaR, a HMM based software that was developed for ChIP-Seq analysis but that in principle can also be used for ATAC-Seq and is probably less sensitive to zero values. Under the constraints of numerous instances of an ‘absence of data’ (Tn5 inaccessible), ChromstaR identified 87 regions that are different between start and control, and stress. All were visually inspected using MACS2 average profiles, normalised by the same number of aligned reads over the genome. Among these 87 regions, ATAC signal was down in stressed samples compared to ‘control and start’ in 45 regions (52%), down in ‘stress and control’ compared to ‘start’ in 16 (18%), up in ‘stress and control’ in 3 (3.4%), and down in ‘control’ in only 1 (1.1%). Seven regions showed a heterogenous pattern on ATAC signals. In 15 regions differences were considered too weak (17%) suggesting that fine tuning of ChromstaR parameters might be necessary in the future. These results are in line with a general decrease in ATAC signal in the stressed samples, *i.e.* chromatin becomes less accessible and/or less heterogenous. It is interesting to note that for 20 regions adjacent ATAC signals (less than 2kb apart) were detected, lending further support to the idea that chromatin structure changes occur in a controlled fashion. Clustering of the samples clearly regroups control and stressed samples ( Figure 7).

### Exposure to Latex leads to differences in chromatin structure between schistosoma adults that developed from exposed (stressed) and unexposed (control) cercaria

**Figure 8.**
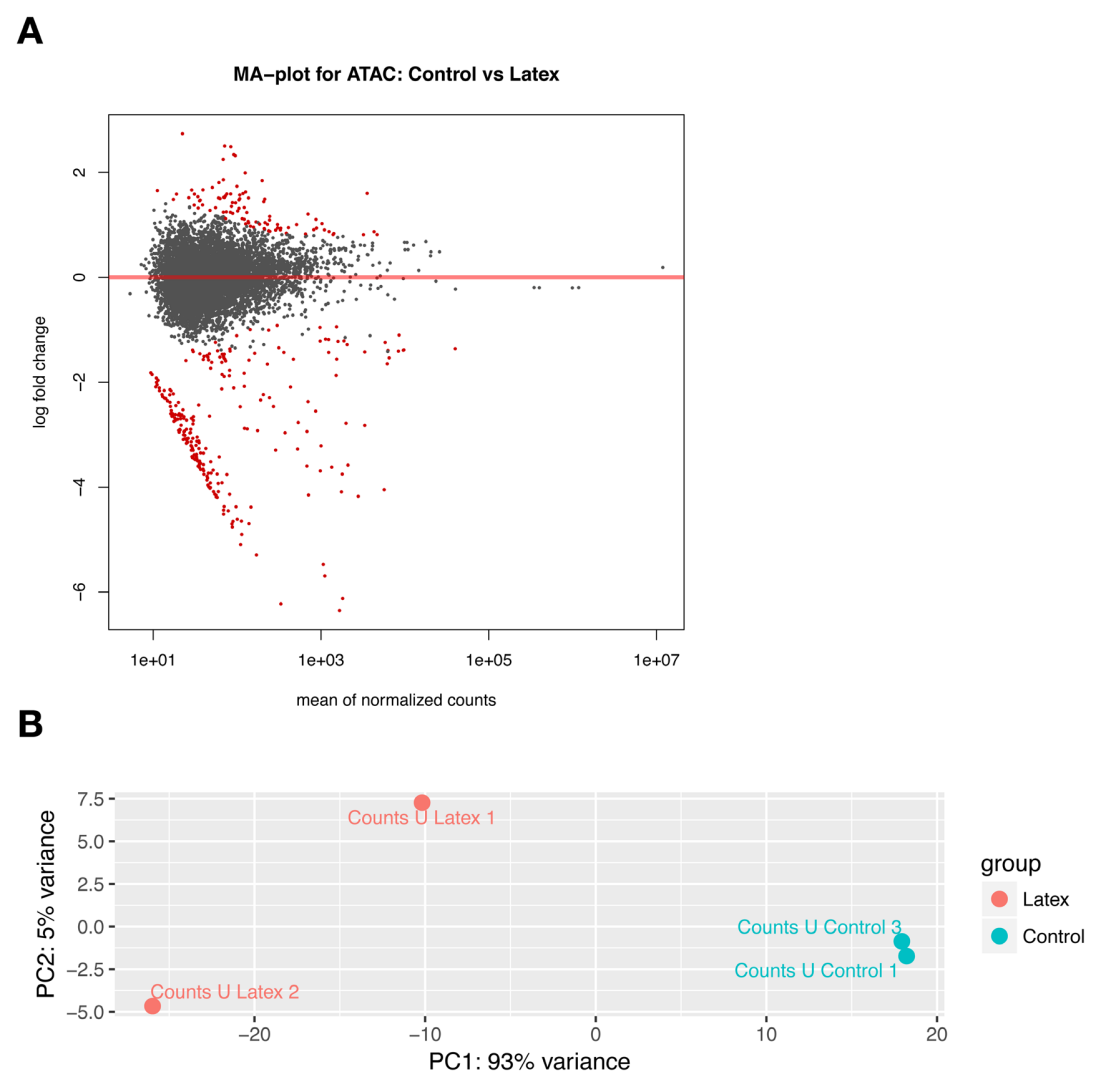
( **A**) MA-Plot and ( **B**) Principal component analysis of individual *schistosoma* based on their ATAC-seq profiles. MA-plots are symmetric. Red dots indicate significantly different ATAC regions in control *vs* “Latex” population. On the PCA every point represents an individual male adult *schistosome*. Populations are colour coded. Samples from the control (blue) and “Latex” population (red) are well separated.

With DESeq2 we found 296 differences between schistosoma adults that developed from latex exposed cercaria and controls with adjusted p-value ≤0.05. MA plots were symmetric around 0 ( Figure 8A), and PCA plots ( Figure 8B) indicated clear segregation of both sample groups. We used in this small study only four worms to demonstrate the feasibility of the method.

## Discussion

Phenotypically, plasticity plays an important role in development and evolution. The relative contribution of genetic and epigenetic components to heritable plasticity is a matter of lively scientific debate ( Hu & Barrett, 2017; Roquis *et al.,* 2018). One of the caveats of analyzing epigenetic information is that it is stored in several, very different bearers of information ( *e.g.* DNA methylation, modification of histones, non-coding RNA and topological position in the interphase nucleus). Nevertheless, these types of information converge towards a change in chromatin structure which can be approximated by DNA accessibility. We reasoned that a straightforward ATAC-seq method to map the chromatin accessibility status in populations with high phenotypic plasticity would facilitate further investigations of the role of epigenetics in plasticity. This study field is also of particular importance to field ecologists. We therefore set-out to establish a robust, easy to use protocol that can be used with little molecular biology training. Our protocol was successfully used in the framework of a summer school ‘Epigénétique en Ecologie et Evolution’ by participants with different levels of expertise in molecular biology using *D. pulex*. We also used single adult *S. mansoni* worms as biological material in a small pilot study. We believe that our protocol is suitable for fast epigenotyping of other organisms as well. From our experience, the only parameter that might be necessary to optimize is Tn5 to chromatin ratio if over- or under-fragmentation occurs. A potential issue is contamination with microorganisms whose DNA might be present in the libraries.

## Data availability

Protocols.io “A simple ATAC-seq protocol for population epigenetics”

https://dx.doi.org/10.17504/protocols.io.bae6ibhe ( Augusto *et al.,* 2019).

A step-by-step protocol for the ATAC-seq procedure

Zenodo: Supporting data for “A simple ATAC-seq protocol for population epigenetics”. http://doi.org/10.5281/zenodo.3828600 ( Augusto *et al.,* 2020).

This project contains the following underlying data:

- Agarose picture (TIF). (Example of electrophoresis fragment separation.)
- Agarose profile (PNG) (Example of fragment separation on a BioAnalyser chip.)
- BioAnalyser, BioAnalyser 2–4 (PDF). (BioAnalyser profiles generated in this study.)
- qPCR cycles (XLSX). (Quantification of qPCR cycles for each daphnia.)
- qPCR plot (JPG). (qPCR amplification cycles plot.)

NCBI SRA:

https://www.ncbi.nlm.nih.gov/bioproject/587385

BioProject Accession PRJNA587385

Data are available under the terms of the Creative Commons Attribution 4.0 International license (CC-BY 4.0).
