# Supplementary material for: A simple ATAC-seq protocol for population epigenetics
Source: Wellcome Open Res. 2021 Jan 7;5:121. Originally published 2020 Jun 9. [Version 2] doi: 10.12688/wellcomeopenres.15552.2 (PMC7814285; doi:10.12688/wellcomeopenres.15552.2)
Supplement: Peer review history

# Peer review: Reviewer response for version 2

**DOI:** 10.21956/wellcomeopenres.18116.r42038

**Published:** 2021-01-18

**Authors:** Frédéric Bantignies

**Affiliations:** 1 Institute of Human Genetics, CNRS, University of Montpellier, Montpellier, France

This new version has been strongly revised. The authors have largely improved their initial manuscript, addressing all my concerns.

 This protocol, that will be useful for population epigenomic studies, is therefore ready for indexing.

Is the rationale for developing the new method (or application) clearly explained?

Partly

Is the description of the method technically sound?

Yes

Are the conclusions about the method and its performance adequately supported by the findings presented in the article?

Partly

If any results are presented, are all the source data underlying the results available to ensure full reproducibility?

Yes

Are sufficient details provided to allow replication of the method development and its use by others?

Partly

Reviewer Expertise:

Genetic and epigenetic, chromatin structure and organization.

I confirm that I have read this submission and believe that I have an appropriate level of expertise to confirm that it is of an acceptable scientific standard.

# Peer review: Reviewer response for version 2

**DOI:** 10.21956/wellcomeopenres.18116.r42037

**Published:** 2021-01-11

**Authors:** José María Santos-Pereira

**Affiliations:** 1 Andalusian Center for Development Biology (CABD), Spanish National Research Council, Pablo de Olavide University, Seville, Spain

The authors have successfully addressed my previous concerns and notably improved their manuscript. Therefore, I find it ready for indexing.

Is the rationale for developing the new method (or application) clearly explained?

Yes

Is the description of the method technically sound?

Partly

Are the conclusions about the method and its performance adequately supported by the findings presented in the article?

No

If any results are presented, are all the source data underlying the results available to ensure full reproducibility?

No

Are sufficient details provided to allow replication of the method development and its use by others?

Partly

Reviewer Expertise:

Chromatin structure, developmental biology, functional genomics.

I confirm that I have read this submission and believe that I have an appropriate level of expertise to confirm that it is of an acceptable scientific standard.

# Peer review: Reviewer response for version 1

**DOI:** 10.21956/wellcomeopenres.17031.r39929

**Published:** 2020-08-17

**Authors:** José María Santos-Pereira

**Affiliations:** 1 Andalusian Center for Development Biology (CABD), Spanish National Research Council, Pablo de Olavide University, Seville, Spain

In this protocol article, Augusto and colleagues describe a version of the popular ATAC-seq method for chromatin accessibility profiling in the invertebrate organisms *Daphnia pulex *and *Schistosoma mansoni*. Their main claim is that this protocol can be used by researchers with little molecular biology training. However, only the wet-lab component of the ATAC-seq experiment is covered by the manuscript, while the computational analysis of the obtained sequences is omitted. I think that this is a major limitation that has to be addressed before indexing (please, see below).

 Major points:

- Using Tn5 transposase with the appropriate number of cells is critical for this protocol. Authors mention that TAGmentation time should be reduced in case that libraries are over-TAGmented. However, they do not mention the cell numbers used with these organisms, nor the number of *Schistosoma* individuals. This is important to be clearly explained in the manuscript.
- The authors claim that this protocol produces robust results. However, the results are not shown at all. A complete ATAC-seq protocol should cover from the sample preparation in the lab to the analysis of the generated results. I think this is a major limitation of this article and that the authors should explain, at least, the primary computational analysis of the data, the performed quality controls and examples of data visualization. An example of basic analysis of the data, such as peak calling and motif enrichment analysis in the called peaks would be much more helpful to understand how this technique may help to study epigenetic diversity at the population level. Of course, these data have to be available for the community, for example by a GEO accession code.

Minor points:

- The origin of the *Daphnia* samples is not explained.

- While I assume that the use of 1% Igepal in the TAGmentation reaction is what lyses the samples, this has to be stated more clearly, since usually cell lysis is performed before TAGmentation in other ATAC-seq protocols.

- The Promega GoTaq G2 polymerase is not a high-fidelity enzyme and therefore I would not recommend its use for this purpose, since this could result in mutations in the amplified molecules and decreased alignment efficiency to the reference genome. Have the authors noted this when analyzing their data in comparison with NEB Next?

- How was the quality of the results obtained by the 13 volunteers? If this protocol can be performed by researchers with little molecular biology training, then the authors should show that the experiments performed by their volunteers were of enough quality to be used.

- The agarose gel showed in the supporting material has no lane labels, so it cannot be known what is shown. It would be helpful to show pictures of the agarose gels underlying Fig. 2 data in the main figure.

Is the rationale for developing the new method (or application) clearly explained?

Yes

Is the description of the method technically sound?

Partly

Are the conclusions about the method and its performance adequately supported by the findings presented in the article?

No

If any results are presented, are all the source data underlying the results available to ensure full reproducibility?

No

Are sufficient details provided to allow replication of the method development and its use by others?

Partly

Reviewer Expertise:

Chromatin structure, developmental biology, functional genomics.

I confirm that I have read this submission and believe that I have an appropriate level of expertise to confirm that it is of an acceptable scientific standard, however I have significant reservations, as outlined above.

# Response

**Published:** 2020-11-18

**Authors:** Christoph Grunau

**Affiliations:** University of Perpignan Via Domitia, France

1- In this protocol article, Augusto and colleagues describe a version of the popular ATAC-seq

 method for chromatin accessibility profiling in the invertebrate organisms *Daphnia pulex *and

*Schistosoma mansoni*. Their main claim is that this protocol can be used by researchers with little

 molecular biology training. However, only the wet-lab component of the ATAC-seq experiment is

 covered by the manuscript, while the computational analysis of the obtained sequences is

 omitted. I think that this is a major limitation that has to be addressed before indexing (please, see

 below).

**It was our initial intention to show the robustness of the wet-bench part. The computational analysis of the obtained sequences is now also provided.**

 Major points:

 2- Using Tn5 transposase with the appropriate number of cells is critical for this protocol.

 Authors mention that TAGmentation time should be reduced in case that libraries are over-

 TAGmented. However, they do not mention the cell numbers used with these organisms,

 nor the number of *Schistosoma *individuals. This is important to be clearly explained in the

 manuscript.

**A sentence describing the number of Schistosoma individuals was added at the animal sampling and transposase mixture section. Number of nuclei were counted and added to the results section, paragraph “ATAC-Seq can be used on individual Daphnia and individual Schistosoma adults”**

 3- The authors claim that this protocol produces robust results. However, the results are not

 shown at all. A complete ATAC-seq protocol should cover from the sample preparation in

 the lab to the analysis of the generated results. I think this is a major limitation of this article

 and that the authors should explain, at least, the primary computational analysis of the

 data, the performed quality controls and examples of data visualization. An example of

 basic analysis of the data, such as peak calling and motif enrichment analysis in the called

 peaks would be much more helpful to understand how this technique may help to study

 epigenetic diversity at the population level. Of course, these data have to be available for

 the community, for example by a GEO accession code

**We have added a data analysis section and two examples of population epigenetics study to show the feasibility.**

**The following sentence was added to the Data availability section: This article contains supporting information online on NCBI SRA (BioProject** **PRJNA587385** **).**

 Minor points

 4-The origin of the *Daphnia *samples is not explained

**This is now in the Animal sampling part.**

 5- While I assume that the use of 1% Igepal in the TAGmentation reaction is what lyses the samples, this has to be stated more clearly, since usually cell lysis is performed before TAGmentation in other ATAC-seq protocols.

**We understand the reviewer’s concern. When AM, RCA and CG optimized the method we realized that adding IGEPAL directly into the tagmentaion reaction did not alter the outcome and that it streamlined the protocol and made it more straightforward**.****

 6- The Promega GoTaq G2 polymerase is not a high-fidelity enzyme and therefore I would not

 recommend its use for this purpose, since this could result in mutations in the amplified

 molecules and decreased alignment efficiency to the reference genome. Have the authors

 noted this when analyzing their data in comparison with NEB Next?

**We did not observe differences between both polymerases following our pipeline. GoTaq is cheaper and that could be an advantage when large number of libraries must be prepared.**

 7- How was the quality of the results obtained by the 13 volunteers? If this protocol can be

 performed by researchers with little molecular biology training, then the authors should

 show that the experiments performed by their volunteers were of enough quality to be

 used.

**As indicated “The protocol described in the methods section was tested by 13 experimenters with molecular biology expert level ranging from untrained to over several 10 years of experience, or some who had retired from active wet-bench work several years ago. In only two cases ATAC-seq library production did not succeed. ” We also added the Figure 7 which shows the clustering of individual *daphnia* based on their ATAC-Seq profiles.**

 8- The agarose gel showed in the supporting material has no lane labels, so it cannot be

 known what is shown. It would be helpful to show pictures of the agarose gels underlying

 Fig. 2 data in the main figure.

**It is our understanding that the supporting data show the raw files. In the Zenode description was added: “In "Agarose picture.Tif" the left lane shows the 100 bp size marker, first 10 bands from down to top: 100bp, 200bp, 300bp, 400bp, 500bp, 600bp, 700bp, 800bp, 900bp and 1kbp.”**

# Peer review: Reviewer response for version 1

**DOI:** 10.21956/wellcomeopenres.17031.r38998

**Published:** 2020-07-21

**Authors:** Frédéric Bantignies

**Affiliations:** 1 Institute of Human Genetics, CNRS, University of Montpellier, Montpellier, France

The authors describe here a simple ATAC-seq protocol for its use in population epigenetics. ATAC-seq is now a common molecular biology technique used to assess chromatin accessibility in nuclei of culture cells or tissues. The authors have adapted this technique to small non-model organisms, which could be a way to determine epigenetic variation/signatures possibly associated with phenotypic variance in animal populations. Although this protocol seems really straightforward and useful for the community (even for non-molecular biologist), several points need to be clarified before its indexing.

**Major points:**

- Would be nice to have an estimation of the size and cell number in both *S.mansoni* and *D.pulex* specimens. This could be important to further adapt this protocol with other organisms of similar size, smaller or larger.
- There is no mention of nuclei isolation or tissue treatment before Transposase treatment. Maybe worth to mention if such steps are needed or not with these organisms, and why.
- There is no mention at all of the sequencing part per se of this ATAC-seq protocol. The protocol closes with “library check”. A paragraph should be added to present the sequencing part of this ATAC-seq protocol. In particular, mention if *D.pulex* and *S.mansoni* genomes are well annotated and/or mention if alternative procedure can be used for sequence data analysis in these cases.
- What would have been very interesting is to provide an illustration that this technique is indeed suitable to quantify epigenetic diversity in populations. For instance, it would have been nice to have examples of a genome browser display emanating from their multiple ATAC-seq libraries in *D.pulex* and/or *S.mansoni*. Maybe, the authors could also refer to some published or ongoing work.

 
**Minor points:**

- Page 2/Introduction: when mentioning *“ATAC-seq uses the Tn5 transposase, which fragments DNA and adds adapters simultaneously in open chromatin regions (that are accessible to Tn5 transposase)”*, the author should further precise where the transposase is cutting the DNA, i.e. in open chromatin, on both sides of nucleosomes and in larger nucleosome free-regions… This would also help to interpret Figure 2 and 3.
- Page 2/Introduction: when mentioning *“Besides their ecological and epidemiological importance, both abovementioned organisms show high phenotypic plasticity in response to environmental cues (e.g. the presence of predator for daphnia) or during their development (schistosoma)”*. For non-ecology specialists, the authors should elaborate a bit more on that, or at least cite references illustrating this intriguing abovementioned phenotypic plasticity in line with associated epigenetic changes.
- Page 2/Introduction/end of the paragraph: I would move *“Controls must be done without organisms as input”* in the Mat&Met/transposase mixture section.
- Page 2/ Mat&Met: I would separate “Animal sampling” and “transposase mixture” sections.
- Page 2/Table2/Reagents produced in the laboratory: I would recommend using commercially available PBS1X, and not sure why another Tagmentation (TD) buffer is indicated here. Usually, the TD buffer is directly provided with the enzyme (as indicated in Table 1). If so, Table 2 is not needed.
- Page 4/Figure1 could be a bit more explicit. At least indicate on the graph the one-third of the maximum fluorescent intensity to determine the additional PCR cycles.
- Page 4/AMPure XP beads double-side purification: “ *This step enriches for the nucleosome-free (~300 bp)*” … “regions as well as di and tri-nucleosome fragments”. Maybe more correct like this. This specific point could be also mentioned in the Figure 3 legend.
- Also mentioned: “ *First transfer 45 ul to an Eppendorf tube (or use PCR tube directly)*”. After the additional PCR cycles and purification, DNA should be in 20 ul elution buffer. Need to be corrected or clarified for consistency in the following of the protocol.
- Page 5/Results: Most data are also presented via a zenodo interface. However, the legends and labels of these figures are not always explicit, and the correspondence with the actual figures of the protocol is not always intuitive, something that could be readily improved.
- Page 6/Figure 3: the figure is showing fragment sizing. All profiles are rather homogeneous, except one, D. Pulex/bottom-left, with two clear peaks, one of small fragments in the range of 35 bp (probably the primers), one of large fragments in the range of 10 kb. As they did for Figure 2, the authors should comment on that and propose an alternative solution when this particular situation is encountered, i.e. needs further purification or it is acceptable to process.

Is the rationale for developing the new method (or application) clearly explained?

Partly

Is the description of the method technically sound?

Yes

Are the conclusions about the method and its performance adequately supported by the findings presented in the article?

Partly

If any results are presented, are all the source data underlying the results available to ensure full reproducibility?

Yes

Are sufficient details provided to allow replication of the method development and its use by others?

Partly

Reviewer Expertise:

Genetic and epigenetic, chromatin structure and organization.

I confirm that I have read this submission and believe that I have an appropriate level of expertise to confirm that it is of an acceptable scientific standard, however I have significant reservations, as outlined above.

# Response

**Published:** 2020-11-18

**Authors:** Christoph Grunau

**Affiliations:** University of Perpignan Via Domitia, France

**Major points:**

 1- Would be nice to have an estimation of the size and cell number in both *S.mansoni *and

*D.pulex *specimens. This could be important to further adapt this protocol with other

 organisms of similar size, smaller or larger

**The number of nuclei were counted in daphnia and schistosoma worms.**

 2- There is no mention of nuclei isolation or tissue treatment before Transposase treatment.

 Maybe worth to mention if such steps are needed or not with these organisms, and why

**We appreciate the reviewer’s concern. This was one of the streamlining steps and in our hands no nuclei isolation was necessary. We tested both approaches. Maybe the small size of the organisms play a role.**

**3- **There is no mention at all of the sequencing part per se of this ATAC-seq protocol. The

 protocol closes with “library check”. A paragraph should be added to present the

 sequencing part of this ATAC-seq protocol. In particular, mention if *D.pulex *and *S.mansoni*

 genomes are well annotated and/or mention if alternative procedure can be used for

 sequence data analysis in these cases.

**The sequencing and analysis parts were added.**

****

**4- **What would have been very interesting is to provide an illustration that this technique is

 indeed suitable to quantify epigenetic diversity in populations. For instance, it would have

 been nice to have examples of a genome browser display emanating from their multiple

 ATAC-seq libraries in *D.pulex *and/or *S.mansoni*. Maybe, the authors could also refer to some

 published or ongoing work.

**We have added the description of the population epigenetics part. We agree that IGV visualisation is definitely useful to get an impression of the data especially in relation to genome annotations. However, we feel that the display is not so useful for the population studies. It was not our intention to use this method to infer any functional relationships between chromatin structure and e.g. gene expression.**

**Minor points:**

 5- Page 2/Introduction: when mentioning *“ATAC-seq uses the Tn5 transposase, which fragments*

*DNA and adds adapters simultaneously in open chromatin regions (that are accessible to Tn5*

*transposase)”*, the author should further precise where the transposase is cutting the DNA,

 i.e. in open chromatin, on both sides of nucleosomes and in larger nucleosome free regions…

 This would also help to interpret Figure 2 and 3

**We modified the sentence as follow:** **“ATAC-seq works similarly as DNase-seq (DNase I hypersensitive sites with high-throughput sequencing) (Song & Crawford 2010), and determines which genomic regions are accessible to Tn5 transposase ( *i.e.* open chromatin regions), presumably the regulatory regions. Tn5 transposase inserts Illumina adapter sequences upon accessing the chromatin, which removes the need for additional steps to make the sequencing libraries later. This simple and efficient protocol reduces the starting material required, compared to DNase-seq. It also avoids many other steps such as the interaction with antibodies ( *e.g.* ChIP-seq) or chemical treatment (e.g. FAIRE-seq, WGBS) that might introduce bias.”**

**6- **Page 2/Introduction: when mentioning *“Besides their ecological and epidemiological*

*importance, both abovementioned organisms show high phenotypic plasticity in response to*

*environmental cues (e.g. the presence of predator for daphnia) or during their development*

*(schistosoma)”*. For non-ecology specialists, the authors should elaborate a bit more on that,

 or at least cite references illustrating this intriguing abovementioned phenotypic plasticity

 in line with associated epigenetic changes

**We added the following sentence: “Classical studies have paved the way to a subsequent rich literature that has documented the amazing property of *Daphnia* to modify their phenotypes at the morphological, physiological, behavioral and more recently at the molecular levels in response to a large panel of environmental stressors including diet, pollution, heavy metals, and predator kairomones (reviewed in (Riessen 2011, Harris et al. 2012)). Furthermore, schistosomes deal with a multitude of signals from the water environment as well as cues that come from their hosts, shaping morphology, metabolism, and infection success in the short-term and also their full development later in life (Roquis et al. 2017, 2018, Augusto et al 2017, 2019). Our group characterized epigenetic mechanisms behind the phenotypic plasticity of schistosomes and their cross-talk with environmental cues, however *Daphnia* phenotype plasticity stills poorly understood.”**

 7- Page 2/Introduction/end of the paragraph: I would move *“Controls must be done without*

*organisms as input” *in the Mat&Met/transposase mixture section.

**This was done.**

 8- Page 2/ Mat&Met: I would separate “Animal sampling” and “transposase mixture” sections.

**This was done.**

 9- Page 2/Table2/Reagents produced in the laboratory: I would recommend using

 commercially available PBS1X, and not sure why another Tagmentation (TD) buffer is

 indicated here. Usually, the TD buffer is directly provided with the enzyme (as indicated in

 Table 1). If so, Table 2 is not needed.

**We understand the reviewer concern, however we would like to keep this information on the paper.  Our group is running several experiments using ATAC-seq and this is a useful information in the case of missing PBS 1X and/or TD buffer. When we use the Illumina kit in the lab, we can often dilute very much the Tn5 enzyme and what gets limiting is the 2xTD buffer. The information about the composition of the buffer is actually not so easy to find and we wish to keep it in the article. We did not see any difference between commercial 2xTD buffer and “home-made” 2xTD. Also, Tn5 can now be purchased separately and even with custom adaptors, which has the potential of even more streamline the protocol.**

 10- Page 4/Figure1 could be a bit more explicit. At least indicate on the graph the one-third of

 the maximum fluorescent intensity to determine the additional PCR cycles.

**The figure was changed (now figure 2).**

 11- Page 4/AMPure XP beads double-side purification: “ *This step enriches for the nucleosome-free*

*(~300 bp)*” … “regions as well as di and tri-nucleosome fragments”. Maybe more correct like

 this. This specific point could be also mentioned in the Figure 3 legend.

**We agree with reviewer suggestion and added the following sentence:** **This step enriches for the nucleosome-free (~300 bp) as well as di and tri-nucleosome fragments**

 12- Also mentioned: “ *First transfer 45 ul to an Eppendorf tube (or use PCR tube directly)*”. After the

 additional PCR cycles and purification, DNA should be in 20 ul elution buffer. Need to be

 corrected or clarified for consistency in the following of the protocol.

**Thank you to have found this! It was a “vestige” of an earlier version of the protocol. Elution is done into 45 µL now and not 20 µL.**

 13- Page 5/Results: Most data are also presented via a zenodo interface. However, the legends

 and labels of these figures are not always explicit, and the correspondence with the actual

 figures of the protocol is not always intuitive, something that could be readily improved.

**We understand the comment. We had provided an Excel file with the description of the supporting raw data. However, this file is not directly accessible. We would need guideline from the editors. Should we provide a detailed description in the manuscript or on the Zenodo depository?**

 14- Page 6/Figure 3: the figure is showing fragment sizing. All profiles are rather homogeneous, except one, D. Pulex/bottom-left, with two clear peaks, one of small fragments in the range of 35 bp (probably the primers), one of large fragments in the range of 10 kb. As they did for Figure 2, the authors should comment on that and propose an alternative solution when this particular situation is encountered, i.e. needs further purification or it is acceptable to process.

**Peaks at 35 bp and 10380 bp are spiked-in marker peaks for the BioAnalyser. They do not come from the samples. This was added to the figure legend.**
